# Dynamic Balance Gait for Walking Assistance Exoskeleton

**DOI:** 10.1155/2018/7847014

**Published:** 2018-07-02

**Authors:** Qiming Chen, Hong Cheng, Chunfeng Yue, Rui Huang, Hongliang Guo

**Affiliations:** Center for Robotics, School of Automation Engineering, University of Electronic Science and Technology of China, Chengdu, China

## Abstract

**Purpose:**

Powered lower-limb exoskeleton has gained considerable interests, since it can help patients with spinal cord injury(SCI) to stand and walk again. Providing walking assistance with SCI patients, most exoskeletons are designed to follow predefined gait trajectories, which makes the patient walk unnaturally and feels uncomfortable. Furthermore, exoskeletons with predefined gait trajectories cannot always maintain balance walking especially when encountering disturbances.

**Design/Methodology/Approach:**

This paper proposed a novel gait planning approach, which aims to provide reliable and balance gait during walking assistance. In this approach, we model the exoskeleton and patient together as a linear inverted pendulum (LIP) and obtain the patients intention through orbital energy diagram. To achieve dynamic gait planning of exoskeleton, the dynamic movement primitive (DMP) is utilized to model the gait trajectory. Meanwhile, the parameters of DMP are updated dynamically during one step, which aims to improve the ability of counteracting external disturbance.

**Findings:**

The proposed approach is validated in a human-exoskeleton simulation platform, and the experimental results show the effectiveness and advantages of the proposed approach.

**Originality/Value:**

We decomposed the issue of obtain dynamic balance gait into three parts: (1) based on the sensory information of exoskeleton, the intention estimator is designed to estimate the intention of taking a step; (2) at the beginning of each step, the discrete gait planner utilized the obtained gait parameters such as step length *S* and step duration *T* and generate the trajectory of swing foot based on (*S*, *T*); (3) during walking process, continuous gait regulator is utilized to adjust the gait generated by discrete gait planner to counteract disturbance.

## 1. Introduction

SCI is a temporary or permanent damage to the spinal cord that changes its function and might cause loss of muscle function and sensation. According to the survey of the World Health Organization [1], between 250,000 and 500,000 people are suffering from SCI every year around the world. SCI patients who are forced to be bedridden and wheelchair bound are susceptible to developing decubitus, loss of bone density, articular contracture of the lower limbs, and deep-vein thrombosis [2]. Gait support using an exoskeleton robot may be an effective way to address the abovementioned problems because a patient wearing the robot moves their legs actively and the ground reaction force stimulates the sensory and musculoskeletal system. Furthermore, the gait support has a particularly meaningful role in the regaining of walking function in several SCI patients. Therefore, lower-limb exoskeletons are designed to provide movement assistance for people suffering SCI and have attracted increasing interest from both academic researchers and industrial entrepreneurs [3–5].

On the development of lower-limb exoskeletons for walking assistance, comfort and safety are two essential features. Many efforts are made on the development of lower exoskeletons for walking assistance. Yan et al.[6] suggest that most of the exoskeletons for assistance still employ predefined trajectories based on off-line simulations or captured human gait data. The generated reference patterns are generally tracked by position controllers of powered joints. It forces patients to move with exoskeletons and lead uncomfortable experience. Moreover, with predefined trajectories, exoskeletons and patients cannot keep balance when encountering a disturbance. Therefore, for obtaining dynamic balance gait, we consider both when to step and how to step.

For when to step, various human-machine interfaces (HMI) are designed. In [7], electromyography (EMG) signal is utilized to get the intention of patients. However, it is difficult to measure EMG signal for patients with SCI motion features such as tilt of torso [8] and upper arms [9] are utilized to obtain the intention of walking and trigger a step. Center of mass (COM) based approaches are also employed in HAL [10] and MINDWALKER [11, 12]. However, these approaches are only based on observed information and the experience of system designer Thresholds in these approaches must be adjusted manually for different patients and situations.

For how to walk, many dynamic gait planning approaches are proposed. In [10], the exoskeleton changes the speed of swing foot dynamically according to duration of support phase for improving the experience of patients. However, balance of exoskeleton is not considered in this research. In [11, 12], extrapolated center of mass (XCoM) [13] method is proposed to prevent the MINDWALKER exoskeleton from falling sideways by online adjusting the step width (hip ab/adduction). However, disturbance in sagittal plane is not taken in account.

In this paper, we proposed a dynamic balance gait approach to obtain balance gait pattern for lower-limb exoskeleton. We model the human-exoskeleton system with linear inverted pendulum (LIP) and design the intention estimator based on the concept of orbital energy diagram. Discrete gait planner (planning a gait at the beginning of each step) and online gait regulator (online adjust the gait during step taking) are proposed for achieving balance gait. The trajectory of swing foot is modeled with DMPs, which can be adjusted dynamically and smoothly. In discrete gait planner, we utilize an optimization method with targeting orbital energy to obtain parameters of a gait trajectory. In online gait regulator, we adjust DMPs dynamically for counteracting the disturbance during a step.

Hence, our contributions are twofolded: first, our approach enables exoskeleton walk as the intention of patients. Secondly, our approach obtain gait trajectories and adjust it dynamically to stabilize balance during walking. Experimental results in simulation environment show the effectiveness and advantages of the proposed method.

## 2. Literature Review

Although predefined trajectory approach is utilized in most of exoskeleton for SCI patients, some attention is paid on technologies for obtain balance gait patterns. In this section, we will lay down the related works about technologies and lower limb exoskeleton systems for keeping balance during walking. Some biomechanics researchers reveal several approaches balance recovery of humanoid robots [14]. These approaches can be divided into two categories: internal joint approach and step taking approach. These approaches also have been used for balance control of exoskeletons.

### 2.1. Internal Joint Approach

Joints of exoskeletons are controlled to serve some crucial features of balance like zero moment point (ZMP) for internal joint approach. In [15], for real-time balance control, variable physical stiffness actuators were implemented to exoskeletons. An abstracted biped model, torsional spring-loaded flywheel, is utilized to capture approximated angular momentum and physical stiffness. The mathematical relation between ZMP and physical stiffness is described with this model. Moreover, ZMP is regarded as conditions of stability. Thus, for keeping balance desired, ZMP is served with the stiffness of joint actuators.

Reference [16] shows that in dynamic balance, the condition for static balance which says the projection of center of mass (CoM) should be within the support polygon is not sufficient and turns out to be instantaneous capture point (ICP) should be within the support polygon. The paper [17] presents a balance control for a powered lower-limb exoskeleton based on the concept ICP and implement it on the exoskeleton named EMY-Balance (CEA-LIST). Joint torques for the specific actuation of EMY-Balance is computed to keep the ICP in support polygon.

In these approaches, ankle joints are needed to be actuated. However, for portability in most of the exoskeletons for SCI patients, ankle joints are not actuated.

### 2.2. Stepping Approach

Stepping approach is widely utilized in balance control for humanoid robots [18, 19] and exoskeletons [11, 12, 20]. MINDWALKER [11, 12] is a powered lower-limb exoskeleton designed for paraplegics to regain locomotion capability. It has five DOFs at each leg, with hip flexion/extension and adduction/abduction and knee flexion/extension powered by SEAs, while hip rotation and ankle pronation/supination passively sustained with certain stiffness. Finite state machine (FSM) is defined with various states and state transitions can also be triggered when the user manipulates the CoM position of the user-exoskeleton system. A trigger to initiate a step will be generated when the projection of the sagittal and lateral CoM positions on the ground fall in the desired quadrant.

To prevent the user-exoskeleton from falling sideways, MINDWALKER implements online correction of the step width by adapting the amount of hip ab/adduction needed during the swing phase. The required adjustment of hip joints is determined using XCoM [13]. If the user-exoskeleton system falls towards one side due to external perturbations such as being pushed at the shoulder or internal perturbations such as user's upper body motion, the foot placement is adjusted resulting in a wider or a narrower step width to counteract such perturbations. However, this XCoM approach does not take the adaptation of sagittal plane in count.

In [20], gait planning for balance is based on ZMP. 7-links model [21] is utilized to model exoskeleton. Trajectories of hip, knee, and ankle are modeled by parameters. These parameters are obtained by optimal algorithm with targeting ZMP. However, this approach is based on the 7-links model which is too complex for exoskeleton.

## 3. Dynamic Balance Gait

In this section, we will introduce the dynamic balance gait approach. We first present the framework of this approach followed by the details of subsystems.

### 3.1. Framework for Dynamic Balance Gait

**Figure 1:**
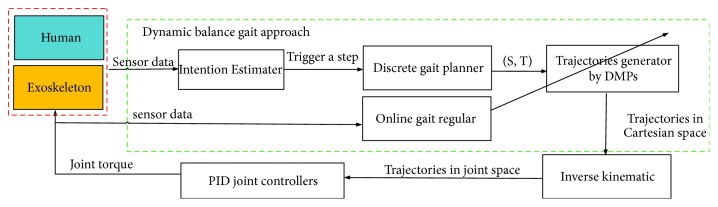
The framework of dynamic balance gait planning.

On the development of lower-limb exoskeletons for SCI patients, most of their ankle joints are passive (without actuators). Therefore, we proposed a novel gait planning approach in this paper which based on the stepping strategy and the LIP model.  Figure 1 shows the framework of proposed dynamic balance gait strategy, which decomposed into three parts: intention estimator, discrete gait planner, and continuous gait regulator.

The gait is divided into single-support phase and double-support phase during normal walking. In single-support phase, the upper body of the human-exoskeleton system is controlled by hip joint of stand leg, which should keep torso stay vertical. Therefore, the gait of human-exoskeleton system can be expressed as foot trajectories of swing leg. We model the trajectories of swing foot with DMPs, which can be learned with sample trajectories of healthy people and adjust gait trajectories online smoothly. Based on the LIP model and gait description with DMPs, three fundamental parts in Figure 1 are employed to achieve dynamic balance gait. According to sensory information of exoskeleton, the intention estimator is designed to estimate the intention of taking a step. At the beginning of each step, the discrete gait planner utilized the obtain trajectory of swing foot. With the intention estimation of taking a step, discrete gait planner obtains gait parameters such as step length *S* and step duration *T*. DMPs are utilized to regenerate trajectories with these different parameters (*S*, *T*). Joints control serve these trajectories to lead exoskeleton and patient move forward. During the process of taking a step, online gait regulator adjusts the parameters (*S*, *T*) based on gait planer to counteract disturbance.

### 3.2. Model of Human and Exoskeleton

**Figure 2:**
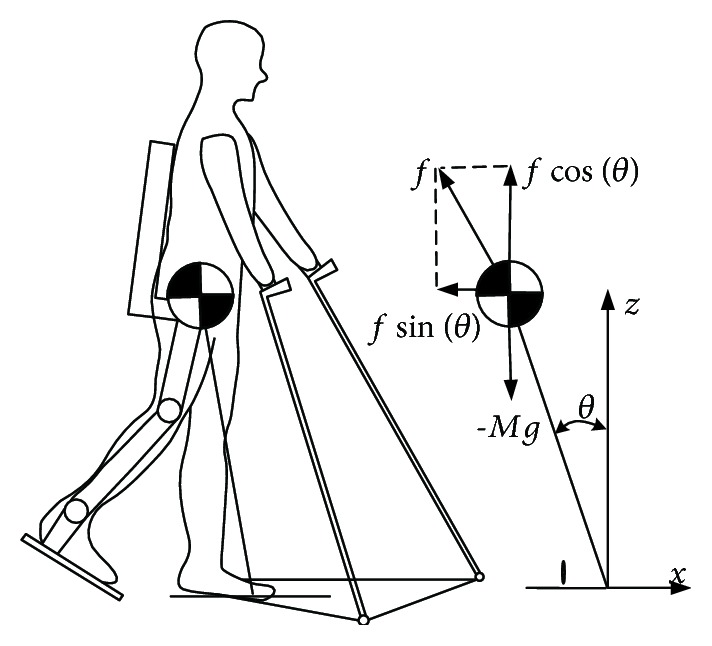
Model of exoskeleton and human based on LIP.

In many applications of exoskeletons, patients walk with crutches to keep balance [8, 9, 11, 12]. Thus, quadruped robot model is utilized to express these human-exoskeleton system. With this model, static stable based on CoM is considered during the walking process. Although patients are enabled to walk again with this approach, for stability, they must rely on crutches, and their gait pattern is less fluent and slower than natural gait. Thus, for achieving fast and natural gait, we use LIP in sagittal plane to model the human-exoskeleton system as Figure 2. Linear inverted pendulum (LIP) is widely used in biped robot [18, 22, 23]. In LIP, we model the body with a point mass with position *r* at the end of a telescoping mechanism (representing the leg), which is in contact with the flat ground. The point mass is kept on a horizontal plane by suitable generalized forces in the mechanism. Most exoskeletons' ankle joints are not actuated and activated [8–10]. Hence, the base of the pendulum can be seen as a point foot, with position *r* ankle. Foot position changes, which occur when a step is taken, are assumed instantaneous and have no instantaneous effect on the position and velocity of the point mass. Patients can apply external force to CoM with crutches to interact with exoskeleton.

By definition mentioned before, we can obtain motion equation of point mass (external force set to 0) as follows:

(1) $$\begin{array}{c} \begin{array}{c} r^{2}\ddot{\theta }+2r\dot{r}\dot{\theta }-gr\sin\left(\theta \right)=\frac{\tau }{M},\\ r^{2}-r{\dot{\theta }}^{2}+g\cos\left(\theta \right)=\frac{f}{M}, \end{array} \end{array}$$

where *r* is the position of point mass (*M*) and *θ* is the angle of LIP with ground. *f* denotes force applied along the LIP. *g* is gravity constants. With *f* = *Mg*/cos(*θ*) and *τ* = 0, the point mass is kept on a horizontal line as $M\ddot{x}=f\sin\left(\theta \right)$. In this situation, motion of point mass can be written as:

(2) $$\begin{array}{c} \ddot{x}=\frac{g}{z}x, \end{array}$$

where *x* and *z* is the position of point mass in *XOZ* plane. Thus, given initial conditions *x*_0_ and ${\dot{x}}_{0}$, we can get the equation motion of CoM as follows:

(3) $$\begin{array}{c} \begin{array}{c} x\left(t\right)=x_{0}\cos h\left(\frac{t}{T_{c}}\right)+T_{c}{\dot{x}}_{0}\sin g\left(\frac{t}{T_{c}}\right),\\ \dot{x}\left(t\right)=\frac{x_{0}}{T_{c}}\sin h\left(\frac{t}{T_{c}}\right)+{\dot{x}}_{0}\cos h\left(\frac{t}{T_{c}}\right), \end{array} \end{array}$$

where *T*_*c*_ is $\sqrt{g/z}$.

### 3.3. Intention Estimator for Taking a Step

Walking with exoskeleton is a periodic phenomenon, and a complete walking cycle is composed of two phases: a double-support phase and a single-support phase. The double-support phase begins with the heel of the forward foot touching the ground and ends with the toe of the rear foot leaving the ground. During the double-support phase, both feet are in contact with the ground. During the single-support phase, one foot is stationary on the ground and the other foot swings from the rear to the front. After the end of a step (swing leg touches the ground), human-exoskeleton system enter double support phase.

**Figure 3:**
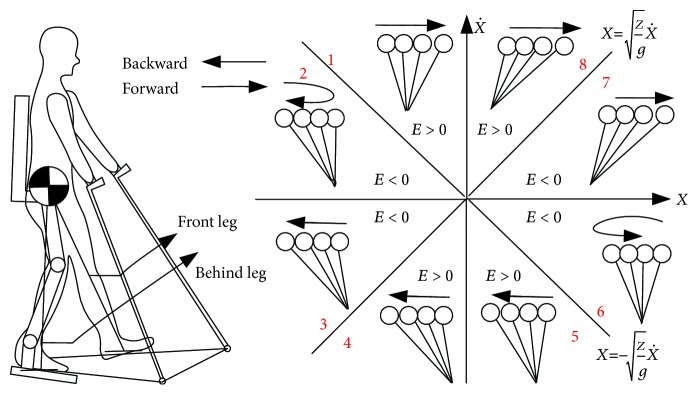
In double-support phase, state of human-exoskeleton system and orbital energy can be describe with the diagram with 8 quadrants.

In this phase, the patient has two choices: stopping to walk and taking a new step. As shown in the left side of Figure 3, the patient moves forward/backward slightly; weight will load on front/behind leg. Thus, we can also use LIP to model this system. With this model, we can design a intention estimator for take a step according to the concept of orbital energy diagram.

The orbital energy *E* [24] can be obtained with the integration of $\dot{x}\left(\ddot{x}-\left(g/z\right)x\right)=0$:

(4) $$\begin{array}{c} \int \dot{x}\ddot{x}-\frac{g}{z}x\dot{x}dt=\frac{1}{2}{\dot{x}}^{2}-\frac{g}{2z}x^{2}=E. \end{array}$$

It is the sum of two terms: dynamic energy and potential energy. It is conserved during a single support phase. Given *E* = 0, LIP moves to straight up position with $\dot{x}=0$. We can infer that if initial *x* > 0, LIP can move over the straight up position with *E* > 0, otherwise it will move back. Thus, letting *E* = 0, we can obtain a line: $x=\pm \sqrt{g/z}\dot{x}$. With axis $\left(x,\dot{x}\right)$ and this line. As shown in Figure 3, we separate the motion states of LIP $\left(x,\dot{x},E\right)$ into 8 regions. Thus, we call it orbital diagram. When *x* < 0 we can get results from orbital diagram:

1. If *E* > 0, *x* < 0 and $\dot{x}>0$, LIP would cross the straight up position.
2. If *E* < 0, *x* < 0 and $\dot{x}>0$, LIP would move back to initial position.
3. If *E* < 0, *x* < 0 and $\dot{x}<0$, LIP would move backward.
4. If *E* > 0, *x* < 0 and $\dot{x}<0$, LIP would move backpack.

As shown in Figure 3, if stand leg is front leg and the state of system can be described with state 1, 7, and 8, then a step must be taken forward to prevent falling down. Commonly, after the transition of weight, system will come to state 1 before 7 and 8. If stand leg is behind leg and the state of system can be described in state 3, 4, and 5, exoskeleton must take a step backward for preventing falling. Thus, after determining stand leg, we can obtain the intention of patient (step forward or step backward) by the quadrant of orbital energy diagram, the status $\left(x,\dot{x},E\right)$ belong to.

### 3.4. Gait Description with DMPs

As many studies on gait planning [25–27] have assumed that the double-support phase is instantaneous, we focus the gait of single-support phase. If foot trajectories and the hip trajectory are already known, all joint trajectories of the exoskeleton can be determined by kinematic equations. The walking pattern can therefore be denoted uniquely by foot trajectories and hip trajectories. As most of exoskeleton ankle joints are not actuated, foot of stand leg can be modeled as point. With known initial position and velocity of LIP, trajectories of hip are known. Thus, gait pattern can be modeled by trajectories of ankle joint of swing leg. In [21], gait pattern is formulated by the constraints of a complete foot trajectory and generate the foot trajectory by third spline interpolation. In [10], min-jert method is used to model this trajectory. However, with these approaches, the whole trajectory must be replanned if a single point of motion changed. In our approach, we obtain the foot trajectory from normal person and regenerate it with targeting step length *S* and duration *T*. DMP is utilized to regenerate this trajectory to adjust this trajectory online.

DMP has been widely employed in robotic applications, since it can solve flexible modelling problems with coupled terms [28]. It is easy to learn with statistical methods and can be adapted through a few parameters after imitation learning [29, 30]. Moreover, it can quickly be adapted to the inevitable perturbations of a dynamically changing, stochastic environment. Modelling a trajectory with the framework of DMP, a trajectory *x*(*t*) is supposed to be the output of a mass spring damper system perturbed by a force term:

(5) $$\begin{array}{c} \tau \dot{v}=K\left(g-x\right)-Dv+\left(g-x_{0}\right)f,\\ \tau \dot{x}=v, \end{array}$$

where *x* and *v* indicate the position and velocity of the system, respectively. *x*_0_ and *g* are the start and goal positions. *τ* is a temporal scaling factor. *K* and *D* are the spring and damping factors of the system. Therefore, with a known *x*(*t*), *f*(*t*) can be calculated through the inverse of the system. Then, *f* can be learned by combining with Gaussian kernels:

(6) $$\begin{array}{c} f\left(s\right)=\frac{\sum_{i=1}^{N}\omega_{i}\psi_{i}\left(s\right)s}{\sum_{i=1}^{N}\psi_{i}\left(s\right)}, \end{array}$$

where *ψ* = exp(−*h*_*i*_(*s* − *o*_*i*_)^2^) are Gaussian basis functions with center *o*_*i*_ and width *h*_*i*_. *w*_*i*_ are weights which should be learned. The phase variable in the nonlinear function (6) is utilized to avoid *f* directly dependence off on time. Use first-order dynamics to define the phase variable *x*:

(7) $$\begin{array}{c} \tau \dot{s}=\alpha s. \end{array}$$

**Figure 4:**
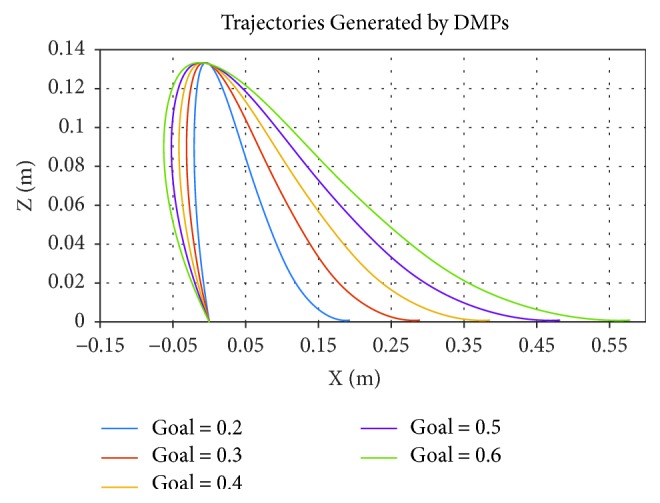
DMPs of foot trajectories with different parameters (*S*, *T*).

The goal *g* is close to the start position *x*_0_, a small change in *g* may lead to huge accelerations, which may reach the limitation of the exoskeleton system. Therefore, modified system equations introduced in [31] are used:

(8) $$\begin{array}{c} \tau \dot{v}=K\left(g-x\right)-Dv+K\left(g-x_{0}\right)s+Kf\left(s\right), \end{array}$$

where the third term can avoid jump movements at the beginning of each step. After obtaining the target function:

(9) $$\begin{array}{c} f_{target}\left(s\right)=\frac{\tau \dot{v}+Dv}{K}-\left(g-x\right)+\left(g-x_{0}\right)s, \end{array}$$

the weighted parameters *ω*_*i*_ are able to learn via statistical learning methods. With specified start position *x*_0_ and goal position *g*, the foot trajectories can be generated through the learn weights *ω*_*i*_. In our approach, we first imitate trajectories of foot of swing leg in single-support phase {*x*(*t*), *z*(t)} with a duration of 1 s. Then, as shown in Figure 4, we regenerate trajectories with different (*S*, *T*) by changing *τ* and *g* by following equations:

(10) $$\begin{array}{c} g_{x}=S,\\ g_{z}=0,\\ \tau_{new}=T\tau_{original}, \end{array}$$

where *τ*_original_ is the time constant of DMP learned before.

### 3.5. Discrete Gait Planner

**Figure 5:**
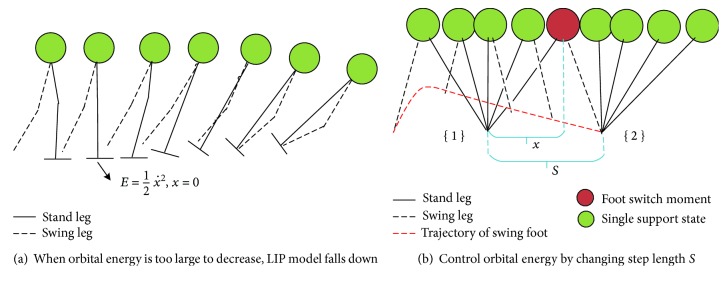
Definition of balance with orbital energy.

Aiming to obtain balance gait, we define the concept of balance based on N-step capturability [18, 32]: the ability of a legged system to come to a stop without falling by taking N or fewer steps. As exoskeleton modeled by LIP, “stop” means orbital energy is 0 ($\dot{x}=0$ when *x* = 0). Thus, the balance of exoskeleton is defined as: the orbital energy can be controlled to 0 with the limitation of swing speed and length of leg. In other words, as shown in Figure 5(a), if *E* is too large to decrease even extending swing leg with max speed, CoM of LIP will go to the limitation of stand leg and body rotate around the tip of toe as shown. However, walking with exoskeleton, patients can change orbital energy by applying external force with crutches. Thus, for walking easily, they expect to walk several steps continuously and smoothly without applying much force on exoskeleton. Therefore orbital energy needs to keep at a positive value.

To control orbital energy, we consider two steps as shown in Figure 5 and consider that the walking gait begins with swing leg leaving the ground and end with contacting the ground. *T* denotes the time cost in this process called gait duration. In the first step, if no external force posed on LIP, we can obtain the *x*_1_(*t*) and ${\dot{x}}_{1}\left(t\right)$ and the *E*_1_ with initial the *x*_1_(0) and ${\dot{x}}_{1}\left(0\right)$. At the moment of leg switching, velocity of CoM does not change (${\dot{x}}_{1}\left(T\right)={\dot{x}}_{2}\left(0\right)$) [22, 23]. Thus, we obtain orbital energy of second step as the following equation:

(11) $$\begin{array}{c} \frac{1}{2}{{\dot{x}}_{1}}^{2}\left(T\right)-\frac{g}{2z}{\left(S-x_{1}\left(T\right)\right)}^{2}=E_{2}, \end{array}$$

where *S* is step length shown in Figure 5(b). With this equation, orbital energy of second step can be controlled by adjusting (*S*, *T*).

Given the aiming orbital energy *E*_2_, the gait planer obtains (*S*, *T*) at each beginning of step. This planner is called discrete gait planner (DGP), since it plans the gait at the beginning of each step.

As the *x*_1_(*t*) and ${\dot{x}}_{1}\left(t\right)$ is the nonlinear function of *t*, we cannot solve it directly. Moreover, for a given targeting *E*_2_, the mount of solutions is infinite. As we learn the trajectory ($\hat{S},\hat{T}$) recorded from healthy person, we expect that the trajectory regenerated by our approach is similar to the original one. Thus, we formulate this problem as optimization problem as follows.

(12) $$\begin{array}{c} \begin{array}{c} \underset{S,T}{argmin}\;J\left(S,T\right)=\alpha {\left(\hat{E}-E\left(T\right)\right)}^{2}+\beta {\left(\hat{S}-S\right)}^{2}+\gamma {\left(\hat{T}-T\right)}^{2}\\ s.t.:\frac{S}{T}<v_{\max},\;S<S_{\max},\;T>T_{\min}, \end{array} \end{array}$$

where $\hat{E}$, $\hat{S}$, and $\hat{T}$ are target values of orbital energy, step length, and duration of gait. *α*, *β*, and *γ* are weighted parameters. Gradient descent method is utilized to solve this optimization problem with gradient as follows:

(13) $$\begin{array}{c} P_{i}=P_{i-1}-\lambda \nabla J\left(S,T\right), \end{array}$$

where *P*_*i*_ = (*S*_*i*_, *T*_*i*_)^2^ and *∇J*(*s*, *t*) is shown as follows:

(14) $$\begin{array}{c} \left(\begin{array}{c} \frac{\partial J}{\partial S}\\ \frac{\partial J}{\partial T} \end{array}\right)=\left(\begin{array}{c} 2\beta \left(S-\hat{S}\right)+\frac{2\alpha g}{z}\left(E\left(T\right)-\hat{E}\right)\left(x\left(T\right)-S\right)\\ 2\alpha \left(E\left(T\right)-\hat{E}\right)\frac{\dot{x}g}{z}\left(x+1\right)+\gamma \left(\hat{T}-T\right) \end{array}\right). \end{array}$$

Iterations of optimization ends with *∇J*(*S*, *T*) = 0 or numbers of iterations reach the limitation. If *∇J*(*S*, *T*) is to 0 after iterations, we obtain the solution (*S*, *T*) closer to the targeting $\hat{E}$ at this step. And *E* will be more closer to target $\hat{E}$ step by step.

### 3.6. Continuous Gait Regulator

As we mentioned, DGP plans a gait at beginning of the step and adjust the orbital energy of the next step. After gait planning, trajectory of foot is fixed during a step. While, if a disturbance occurs during single-support phase, the gait cannot change until leg switching moment. Thus, we design a continuous gait regulator (CGR) to adjust the gait continuously during swing phase.

CGR adjusts gait by changing the parameters (*S*, *T*) obtained from discrete gait planner. It improve the DGP's ability of keeping balance. In each sample time *i*, we can obtain the $\dot{x}\left(i\right)$ and *x*(*i*). We calculate (*S*_*i*_, *T*_*i*_) with the same optimal approach of DGP and update remain time *T*_remain_ of original trajectory generated by DGP. Then, we change the parameter of the DMPs with *g* = *S*_*i*_ and *τ* = *T*_remain_/*T*_*i*_.

## 4. Experiments on Simulation

In this section, we first lay down on the performance metrics definition and evaluate this approach in simulation platform.

### 4.1. Performance Metrics Definition

Evaluating the performance of proposed approach is to evaluate the ability of keeping balance especially when a disturbance occurs. As we mentioned before, balance of system is depended on the controllable ability of orbital energy. Thus, in our evaluation, we exert disturbance and compare orbital energy of different approaches during the whole process.

### 4.2. Simulator Introduction

**Figure 6:**
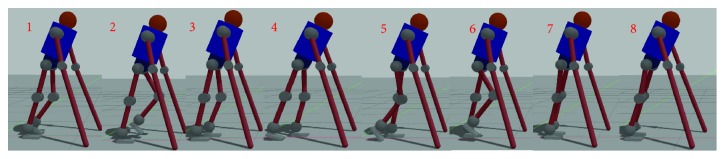
Snapshot of simulation during walking.

We build a simulator to evaluate the performance of our approach in a desktop-computing platform with CPU:i7 4790 k and 8 G RAM with Gazebo robotics simulation software as shown in Figure 6. As the patient holds crutches to preserve falling sideways during normal walking, we model this coupled system as model shown in Figure 6 and constrain the motion in sagittal plane. In this model, hip joint (flexion/extension) and knee joint (flexion/extension) are actuated just like most of exoskeletons. PID controllers with 1000 Hz sample frequency are used in each joint with limitation of output torque 200 N.M. At each joint, joint encoders are embedded to obtain motion state of joints. We simulate 3000 ms in each trail with initial state of LIP: $x_{init}=0.1\;m,{\dot{x}}_{init}=0.1\;m/s,z=0.7\;m$. The targeting orbital energy is set to be 0.4 J. Four gait patterns are compared in our experiment:

Gait with fixed parameters: (*S* = 0.35, *T* = 0.25).

Gait with fixed parameters: (*S* = 0.3, *T* = 0.25).

Gait generated by DGP.

Gait generated by DGP combined with CGR.

Different disturbance: −5 N, −15 N, −25 N, 5 N, 15 N, and 25 N in sagittal plane is posed on LIP from 500 ms to 2000 ms. A trail ends if the model falls down during walking. Orbital energy and foot transition of different gait patterns are recorded for evaluating the ability to keep balance.

### 4.3. Experiment Results

**Figure 7:**
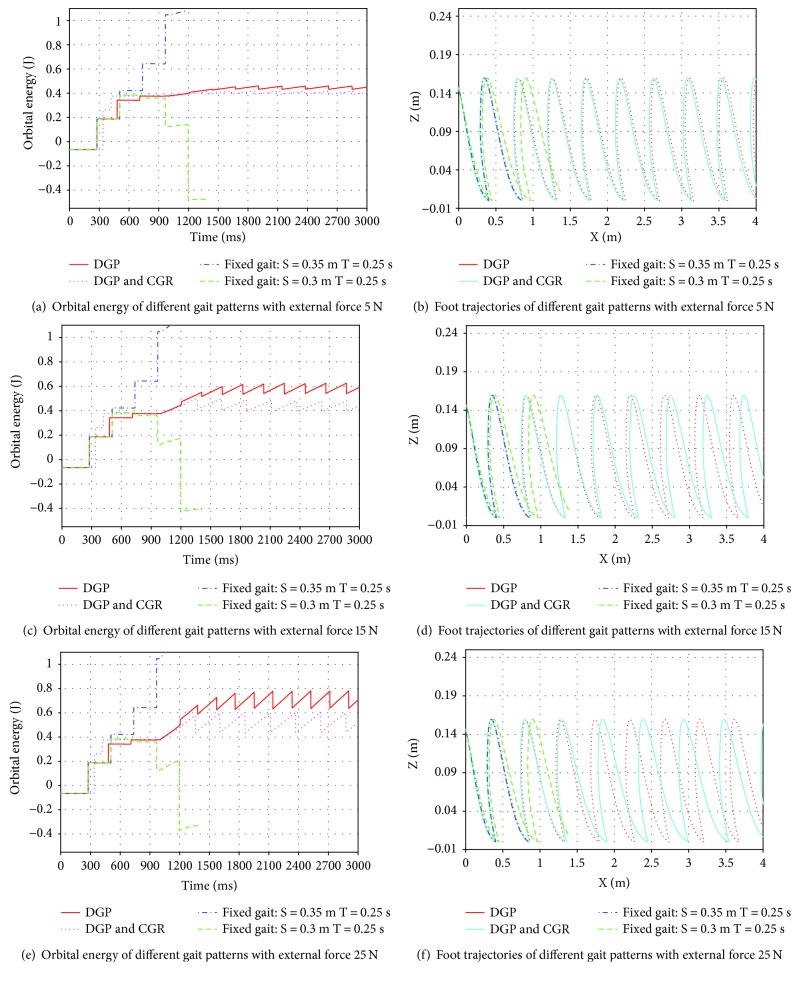
Experimental results of different gait patterns with external force (5 N, 15 N, 25 N).

Figure 7 illustrates the simulation results of different gait patterns with positive external force as disturbance: 5 N, 15 N, and 25 N. With fixed gait pattern, the model of human-exoskeleton in simulation falls down forward after 5 steps. Patients have to control body with stick to keep balance with fixed gait. Both DGP and DGP combining with CGR can keep balance with disturbance from 5 N to 15 N. As disturbance increases, the error orbital energy of DGP increases significantly. With DGP alone, orbital energy of system cannot get back to given target after step taking. As shown in Figure 7(f), trajectory of swing foot trajectory generated by DGP combined with CGR adjusts the step length after encountering a disturbance. Thus, DGP combined with CGR achieves better performance than DGP in orbital energy control.

**Figure 8:**
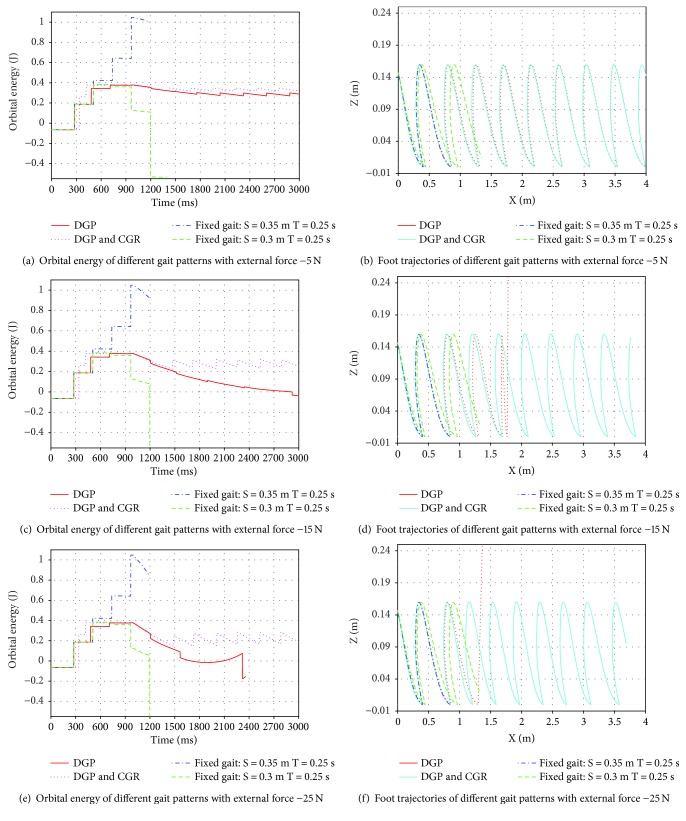
Experimental results of different gait patterns with external force (−5 N, −15 N, and −25 N).

Simulation results with native external force as disturbance is shown in Figure 8. Walking with fixed gait model, the patient falls down without providing force to keep balance. With external force −5 N, gait generated by both DGP and DGP with CGR can achieve balance during the whole process. However, as shown in Figure 8(d) and 8(f), model will fall down with the external force increasing to −15 N. Compared to DGP, DGP with OGR can still achieve balance with disturbance (−25 N) and control the orbital energy close given value.

## 5. Conclusions and Future Works

In this paper, we proposed a novel approach to obtain dynamic balance gait. We model exoskeleton-human system with LIP and express gait trajectory with DMP. Three subsystems intention estimator, discrete gait planner, and continuous gait regulator, are designed. Intention estimator is designed based on the orbital energy diagram to get the intention to step forward or backward. With the intention of patient, discrete gait planner obtain the gait parameters (*S*, *T*) to keep balance. To improve the ability to counteract disturbance, continuous gait regulator is designed to change gait in time. Experiments on both simulation and real system with different environment demonstrate the efficiency of this approach.

In the future, we will firstly extend this approach to different environments. For example, external force would let the system lose balance during walking process, which is unacceptable. LIP should be modified with the changing height of model for upstairs walking situation. Then the ankle joints of exoskeletons for SCI patients are always passively actuated. Thus, LIP model with point foot is used to model exoskeleton-human system. However, spring damping system are employed in exoskeletons which should be taken into account.

## Data Availability

Readers can access the data supporting this study by the clone git of this program: “https://gitee.com/kipochen_uestc/LIP_python.git” or from the corresponding author upon request.
